# Role of Selenium and Selenoproteins in Male Reproductive Function: A Review of Past and Present Evidences

**DOI:** 10.3390/antiox8080268

**Published:** 2019-08-02

**Authors:** Izhar Hyder Qazi, Christiana Angel, Haoxuan Yang, Evangelos Zoidis, Bo Pan, Zhenzheng Wu, Zhang Ming, Chang-Jun Zeng, Qingyong Meng, Hongbing Han, Guangbin Zhou

**Affiliations:** 1 Farm Animal Genetic Resources Exploration and Innovation Key Laboratory of Sichuan Province, College of Animal Science and Technology, Sichuan Agricultural University, Chengdu 611130, China; 2 Department of Veterinary Anatomy & Histology, Shaheed Benazir Bhutto University of Veterinary and Animal Sciences, Sakrand-67210, Sindh, Pakistan; 3 Department of Veterinary Parasitology, College of Veterinary Medicine, Sichuan Agricultural University, Chengdu 611130, China; 4 Department of Veterinary Parasitology, Faculty of Veterinary Sciences, Shaheed Benazir Bhutto University of Veterinary and Animal Sciences, Sakrand-67210, Sindh, Pakistan; 5 Department of Nutritional Physiology and Feeding, Faculty of Animal Science and Aquaculture, Agricultural University of Athens, 75 Iera Odos, 11855 Athens, Greece; 6 State Key Laboratory of AgroBiotechnology, China Agricultural University, Beijing 100193, China; 7 National Engineering Laboratory for Animal Breeding, Key Laboratory of Animal Genetics and Breeding of the Ministry of Agriculture, Beijing Key Laboratory for Animal Genetic Improvement, College of Animal Science and Technology, China Agricultural University, Beijing 100193, China

**Keywords:** male fertility, mammalian reproduction, selenium, selenoproteins, spermatogenesis

## Abstract

Selenium (Se) is an important trace mineral having many essential roles at the cellular and organismal levels in animal and human health. The biological effects of Se are mainly carried out by selenoproteins (encoded by 25 genes in humans and 24 in mice). As an essential component of selenoproteins, Se performs structural and enzymic roles; in the latter context it is well known for its catalytic and antioxidative functions. Studies involving different animal models have added great value to our understanding regarding the potential implications of Se and selenoproteins in mammalian fertility and reproduction. In this review, we highlight the implications of selenoproteins in male fertility and reproduction followed by the characteristic biological functions of Se and selenoproteins associated with overall male reproductive function. It is evident from observations of past studies (both animal and human) that Se is essentially required for spermatogenesis and male fertility, presumably because of its vital role in modulation of antioxidant defense mechanisms and other essential biological pathways and redox sensitive transcription factors. However, bearing in mind the evidences from mainstream literature, it is also advisable to perform more studies focusing on the elucidation of additional roles played by the peculiar and canonical selenoproteins i.e., glutathione peroxidase 4 (GPX4) and selenoprotein P (SELENOP) in the male reproductive functions. Nevertheless, search for the elucidation of additional putative mechanisms potentially modulated by other biologically relevant selenoproteins should also be included in the scope of future studies. However, as for the implication of Se in fertility and reproduction in men, though a few clinical trials explore the effects of Se supplementation on male fertility, due to inconsistencies in the recruitment of subjects and heterogeneity of designs, the comparison of such studies is still complicated and less clear. Therefore, further research focused on the roles of Se and selenoproteins is awaited for validating the evidences at hand and outlining any therapeutic schemes intended for improving male fertility. As such, new dimensions could be added to the subject of male fertility and Se supplementation.

## 1. Introduction

### 1.1. Background

In 1817, Se was first identified by Jöns Jacob Berzelius, who was conducting an investigation on chemicals responsible for the outbreaks of ill health amongst the workers in a sulfuric acid plant in Sweden [1]. Its basic importance in both normal growth and reproduction in animals was not discovered until the 1950s [2]. However, the definitive manifestation for the importance of Se in humans came through the findings of large-scale trials in China that demonstrated the ameliorative effects of Se supplementation on children and young adults suffering from the Keshan’s disease (characterized by cardiomyopathy); once endemic in areas with low soil Se levels [3,4].

### 1.2. Implication of Se in Mammalian Reproduction: An Overview

The optimal reproductive efficiency of mammals depends on many factors, such as genetics, nutrition, management, and environmental determinants [5]. Among these, trace mineral nutrition is vital for various biological functions such as, normal growth, development, and reproduction [6]. Furthermore, even the narrow variations in the levels of micronutrients (such as trace minerals) may have considerable bearing on vital biological processes including reproductive health and performance [5,6]. Similarly, as for the Se, there is a constringe window between inadequacy and nimiety and its essential and toxic levels are clearly defined [7,8]. In order to sustain the optimum Se concentrations within cells, enormously controlled mechanisms should be in place [8]. Ideally, for investigating whether or not the Se supplementation could ameliorate the fertility, the reference range for Se adequacy/inadequacy should be pre-defined [7]. The Se concentration, for the most part, depends on the tissue being analyzed [7]. As a matter of fact, it is also unclear which biological fluid viz. blood, serum, seminal plasma, sperm cells, follicular fluid provides the most precise picture of Se concentration with regards to its role in reproduction [7]. Apparently, there is a dearth of scientific information elucidating the relation between serum Se and the levels in the reproductive tissues [7]. Nevertheless, few past reports on mammalian models have demonstrated the relationship between Se status and reproductive performance in both males [5,9] and females [5,10]. The adequate traces of Se in the male reproductive organs are essentially required for normal spermatogenesis, sperm maturation, and sperm motility and overall function [11,12,13,14]. Increased dietary Se intake has also been implicated in enhancing the antioxidant glutathione peroxidase (GPX) activity, thereby improving fertility in male [15].

### 1.3. Selenium Biochemistry and Significance

**Figure 1:**
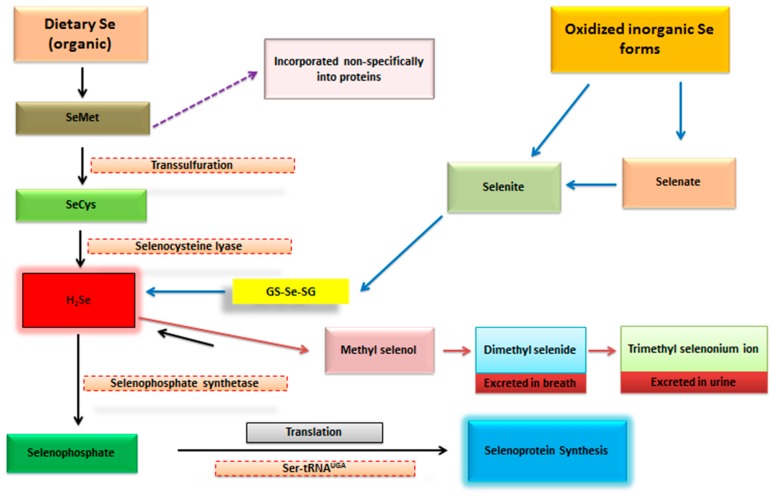
Metabolic pathways of different forms of selenium. Selenomethionine (SeMet) from organic sources (Se-yeast) and other food proteins undergoes transsulfuration reactions and is converted to selenocysteine (SeCys). Selenocysteine is then converted to hydrogen selenide (H_2_Se); this reaction is catalyzed by a substrate-specific enzyme selenocysteine lyase. Hydrogen selenide is converted to selenophosphate via selenophosphate synthetase, and following reaction with tRNA-bound serinyl residues, it produces a SeCys-bound tRNA from which SeCys is inserted co-translationally and further translated to selenoproteins (GPX, SELENOP etc.). Hydrogen selenide could also be methylated and detoxified, and excreted in breath (as dimethyl selenide) and urine (as trimethyl selenonium ion). Alternatively, selenomethionine may also be non-specifically incorporated into proteins such as albumin and hemoglobin in place of methionine. Inorganic forms of Se such as selenate and selenite are metabolized through thiol-dependent reduction reactions, producing hydrogen selenide, which is a starting point for the synthesis of selenoproteins as delineated earlier for organic forms [22,23]. Definitions: GS-Se-SG: selenodiglutathione; GPX: glutathione peroxidase; H_2_Se: hydrogen selenide; Se: selenium; SeMet: selenomethionine; SeCys: selenocysteine; SELENOP: selenoprotein P; Ser-tRNA^UGA^: seryl tRNA (also Sec-tRNA^[Ser] Sec^), and the specific in-frame stop codon (UGA) present in the selenoprotein messenger RNAs.

Generally, Se is utilized in organic or inorganic forms. Previously, it has been discussed that form of Se has an important bearing on its possible ameliorative effects and/or adverse toxicological consequences on general wellbeing and development of organisms [8,16,17]. In addition, it has also been argued that both form and the total intake of Se are equally important with regards to the potential health-related effects. However, it should be emphasized that form of Se consumed might be of more value in this context [18]. It has been reported that organic forms of Se have greater bioavailability compared to the inorganic forms [19]. Selenium from organic sources is assimilated much more efficiently than the inorganic forms, and could be expeditiously utilized for synthesis of selenoproteins in conditions of stress [20,21]. Besides, the toxicity level of organic forms is also lower than that of inorganic selenite or selenate species, indicating that the higher bioavailability may be compensated by lower toxicity levels [22]. Moreover, it has been demonstrated that for modulation of antioxidant activities of GPXs under oxidative stress conditions, selenocysteine, derived from selenomethionine following B_6_-dependent-transsulfuration reactions, in organic Se is mineralized to selenide and re-synthesized into a seleno-cysteinyl moiety that regulates the gene expression and activates the GPX system. In contrast, the metabolic pathway of inorganic Se is dissimilar i.e., it bypasses the B_6_-dependent-transsulfuration process for the modulation of GPX system [23,24,25]. Recently, such aspects regarding the synthesis and metabolism of dietary Se forms have been comprehensively reviewed [18,21], and the bioavailability of different Se species is also reviewed in greater detail elsewhere [26]. Brief description of metabolic fate of different forms of Se is depicted in Figure 1. For ready reference, different forms of Se and their characteristics are briefly summarized in Table 1. Meanwhile, recommended dietary allowances (RDAs) of Se for human and different animal species are presented in Table 2.

**Figure 2:**
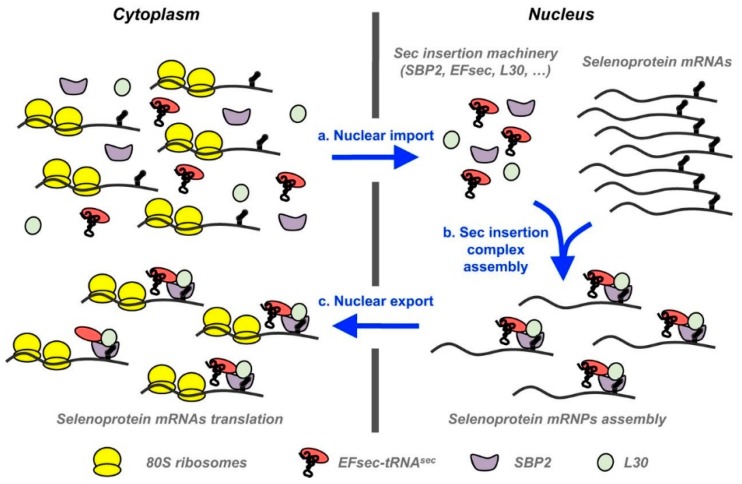
Schematic of the nuclear selenoprotein mRNPs assembly. “Recoding factors are imported into the nucleus to assemble with selenoprotein mRNAs. mRNPs are built and exported to the cytoplasm to promote efficient translation by the ribosome. An increase in nucleocytoplasmic shuttling in response to H_2_O_2_ induced oxidative stress is proposed to improve selenocysteine insertion complex nuclear assembly and, consequently, selenoprotein expression”. Credit lines: “Republished with permission of *Journal of Biological Chemistry*, American Society for Biochemistry and Molecular Biology, from [49]; permission conveyed through Copyright Clearance Center, Inc”. Definitions: mRNP: Messenger ribonucleoprotein; Sec-tRNA^sec^: tRNA bearing the anticodon for UGA with selenocysteine attached; EFsec: elongation factor specific for Sec-tRNA^sec^; SBP2: SECIS-binding protein 2; L30: a ribosomal protein. Note: In addition to these components of SECIS elements, eukaryotic translation initiation factor (eIF4a3), and nucleolin are also considered as the essential factors which are needed for Sec incorporation into proteins in response to the UGA codon [46].

As an important micronutrient, Se has many essential roles at the cellular and organismal levels in animal and human health, and relevancy to various patho-physiological conditions [27,43,44]. Both Se deficiency and excess have been demonstrated to impact the redox status and result in adverse health outcomes in animals and human beings [45]. The biological functions of Se are mainly carried out by selenoproteins which are demonstrated in all three domains of life [43,46]. As an essential component of selenoproteins, Se performs structural and enzymic roles; in the latter context it is well known for its catalytic and antioxidative functions [43,44]. All selenoproteins contain at least one selenocysteine (Sec), a Se-containing amino acid (the 21st “naturally occurring” amino acid) [43,47,48], which is co-translationally infixed into the incipient polypeptide chains in response to the UGA codon, which usually functions to end the translation process [46]. It is worthwhile to mention that selenoprotein messenger RNAs (mRNAs) have two distinct attributes, which are absent in other mRNAs. One of those distinct features is the presence of UGA in the open reading frame (ORF), and other one is the selenocysteine insertion sequence (SECIS) element (stem-loop structure) in the 3′ untranslated region (3′ UTRs) of mRNA [19]. This SECIS element is involved in controlling the proper recoding of the UGA codon as selenocysteine, and thus, precluding the synthesis of truncated proteins [49]. Nevertheless, synthesis of selenoproteins is still a complex process and because its regulation is mediated by many variable parts, it remains incompletely understood [19,46]. Intriguingly, it has been suggested that multiple SECIS-binding proteins might also have implication in regulating the expression of selenoproteins in a combinatory pattern. Therefore, more research is required to elucidate the physiological function of different SECIS-binding proteins in controlling the hierarchy of selenoprotein expression [46]. The steps involved in nuclear selenoprotein mRNPs (messenger ribonucleoprotein) assembly and transport are depicted in Figure 2.

Recently, it has been demonstrated that expression of selenoproteins in mammals and in vitro cultured cells is modulated by Se levels [19,49]. This regulation occurs mostly during the translation phase, and moderately at mRNA transcription levels. This selective regulation of expression ensures that essential selenoproteins are maintained at the expense of others [19,49]. Interestingly, in conditions of insufficient or low Se provision, the synthesis of certain selenoproteins such as, glutathione peroxidase 4 (GPX4) is prioritized over that of others [50]. Conversely, glutathione peroxidase 1 (GPX1) is regarded as one of the most highly sensitive selenoproteins (ranks in the bottom of selenoprotein hierarchy (reviewed in [19])) to the alterations in Se status, with levels of mRNA and protein dramatically decreased under low Se conditions [51]. In these conditions, Gpx1 is downregulated (freeing Se) so that selenoprotein P (Selenop) could be maintained for transporting Se to other tissues and cells [19]. In Se-deficient conditions, reduced expression of selenoproteins is likely due to the alterations in rate of mRNA turnover due to nonsense-mediated decay rather than the transcriptional regulation. It is also believed that Sec incorporation is a limiting step in synthesis of selenoproteins, which, along with nonsense-mediated decay, is mainly responsible for the regulation of selenoprotein expression by dietary Se [46]. Besides, it should be noted that, at present, the interplay between Se status and other confounding factors is also poorly studied, particularly; there is an apparent dearth of research on selenoprotein regulation by oxidative stress or in Se-rich conditions [52]. Recently, it has been demonstrated that expression of certain antioxidant selenoproteins is also modulated by oxidative stress in conditions of limiting Se [49]. Intriguingly, it has been reported that increased levels of reactive oxygen species (ROS), endoplasmic reticulum (ER) stress and oxidation-mediated DNA damage are the expected events in situations of downregulated expression of selenoproteins, leading towards the impaired cellular homeostasis and perturbations in progression of cell cycle. In conditions of Se supplementation, expression of certain selenoproteins is increased and might play an essential role in the efficient counteracting of harmful levels of ROS in oxidative stress-related challenges [49].

Among others, SELENOP has the special mention, as it contains more than one Sec [19], and being a major plasma selenoprotein, it delivers Se primarily from the liver to several other organs [53,54], and is involved in Se transport and metabolism within organs [19,47]. Like most selenoproteins with known functions, SELENOP also bears an *N*-terminal Sec-containing thioredoxin functional domain, which is indicative of its potential redox function. Many other selenoproteins such as, selenoprotein H (SELENOH), selenoprotein T (SELENOT), selenoprotein V (SELENOV), and selenoprotein W (SELENOH), also have thioredoxin like domains, designating redox-related roles [19,55]. However, the individual characteristics and biological roles of several selenoproteins still remain mostly unidentified [27,43,56]. In recent past, a significant progress has been achieved in characterizing selenoproteins and interpreting their physiological roles [46]. However, further functional characterization of selenoproteins and selenoproteomic analyses may explicate the different biological roles of Se and help in the identification of novel molecular pathways and biological processes that are dependent on selenoproteins [46].

### 1.4. Rationale

In past decade, many studies and a few literature reviews have reported a strong implication of Se and selenoproteins in mammalian reproduction [14,43]. However, there is still a significant need for bringing together the new research findings and critical thinking and condense the information for better understating of Se-functions in male reproduction. Therefore, in this review, with elaborated appraisal of the related studies (both animal and human) published till date (2019), we describe the implications of Se and selenoproteins in male reproductive function, related disease conditions and experimentally-induced challenges.

## 2. Important Selenoproteins Relevant to Male Reproduction

Eight different enzymatic isoforms of glutathione peroxidase (GPX) family (GPX 1–8) play a vital role in several redox responses. Of note, GPX1 to 4 and GPX6 are selenoproteins, while the other isoforms are Cys-containing analogs instead. Moreover, GPX6 is the lone exception and a selenoprotein in man [57]. The principal role of these enzymatic isoforms is to safeguard and protect the cells from oxidative stress by catalyzing the reduction (via glutathione) of hydrogen peroxide (H_2_O_2_), organic hydroperoxides and lipid peroxides. All these are tissue specific and apparently have a sexually dimorphic expression [58].

Glutathione peroxidase 4, also known as PHGPx (phospholipid hydroperoxide GSH Px), is a classical case. It is distinctly expressed in testes and has both an antioxidant as well as a structural role; the latter context is evident from a fact that it constitutes over 50% of mitochondrial capsule (as an oxidatively inactivated protein) in midpiece of mature sperm [9]. In an early stage of spermatogenesis, GPX4 is believed to protect the developing sperm from oxidative stress-induced DNA damage, however, in the later phase, through cross linkage with proteins in midpiece region, it provides the integrity to the sperm midpiece by becoming a structural component of mitochondrial sheath circumventing the flagellum, which is an essential component for sperm stability and motility [59,60,61,62]. Three isoforms with different N-terminal amino acid sequences viz. mitochondrial (mGPX4) and cytosolic (cGPX4) and nuclear variant (sperm nucleus glutathione peroxidase 4, snGPX4) are encoded by a single *GPX4* gene. These three isoforms are specifically localized in the mitochondria, cytosol, and nucleus [63]. Following complete targeted disruption of *Gpx4*, early embryonic lethality in homozygous *Gpx4* knockout (KO) mice has been witnessed; however, on the other hand, in spite of their decreased Gpx4 mRNA and protein levels, the heterozygote mice were viable, fertile and appeared normal [63]. Conrad et al. [64] have showed that mice with a directed deletion of sn*Gpx4* gene were not only viable, but also fully fertile, in contrast to the full KO mice. The deletion of *cGpx4* variant results in the early developmental defects (reviewed in [65]). On the other hand, Schneider et al. [66] have demonstrated that normal embryonic and postnatal development could be achieved even after targeted deletion of m*Gpx4* in mouse model; however, this situation leads to infertility in males. Interestingly, resulting infertility was bypassed following intra-cytoplasmic sperm injection of *mGpx4−/−* sperm and viable offspring were produced [66]. Imai and co-researchers [67] demonstrated the importance of GPX4 for male fertility in humans. They found that GPX4 was abundantly distributed in late spermatocytes and spermatids and localized in the sperm midpiece, particularly in the mitochondria. These authors also report that, among examined subjects, all but 10% of the infertile men demonstrated a striking decline in GPX4 levels in sperm. However, no abnormal expression of GPX4 was observed in sperm from fertile males [67]. These results were, however, confirmed by Foresta and colleagues [9] who reported that both sperm structural integrity and motility were directly related with the concentration of GPX4. With the same token, residual GPX4 activity was significantly reduced in infertile men compared to the controls, and particularly reduced in oligoasthenozoospermic men. This activity showed a direct correlation, most importantly, with the forward motility, but also with the viability and structural integrity [9]. In another study on murine model in 2009, it was further demonstrated that reduced Gpx4 levels lead towards infertility in males [68]. This was further supported by the evidence that *mGpx4* KO epididymal sperm were unable to fertilize the oocytes in vitro [68]. Therefore, regardless of cause of reduced GPX4, it might be regarded as the prognostic tool for determining the fertility [9]. In vitro fertilization (IVF) studies have also been extended to assess the general impact of sperm GPX expression on fertilization ability, embryo quality and over all reproductive outcomes. More asymmetric embryos (at day 3) were obtained from sperm samples with a lower *GPX4* mRNA expression. However, *GPX4* levels showed no effect on the later phase of in vitro development (at days 5 and 6) or on pregnancy rates [69].

In addition to these evidences, recently Parillo and colleagues [70] reported that strong expression signals of GPX4 protein were observed in seminiferous tubules (apical region) of healthy Chianina bulls, where it was localized in the cytoplasmic region of maturing sperm i.e., spermatogonia, round spermatids, and elongated spermatids. Similarly, immuno-signals were also observed in the epididymal and ejaculated sperm particularly in acrosome region. These dynamic strong signals at different stages of sperm maturation conform to the earlier biochemical observations indicating that GPX4 is essential for the optimal development and function of bovine sperm similar to other mammalian species [70].

Like GPX4, SELENOP is also believed to play an essential role in male reproductive functions. It serves as a transport protein for Se and is also expressed in vesicle like structures in the basal region of the Sertoli cells [71]. Besides, *Selenop* mRNA was also expressed in Leydig cells of rats (discussed later in 3.1). Noticeable reduction in fertility, reduced levels of Se and a lower Gpx activity have been reported in male *Selenop* KO mice [72]. Meanwhile, a diet containing high Se content could not restitute the testis Se levels or normal sperm phenotype in these mice [71]. X-ray fluorescence microscopic observations in *mGpx4* KO mice models have revealed a 60% decline in Se levels, and a resultant greatly impaired spermatogenesis. However, the largest loss of Se was manifested in *Selenop* KO models; in which a 77% decrease of Se was observed [73]. In addition to these two peculiar and canonical selenoproteins (GPX4, SELENOP), other selenoproteins such as GPX1 and GPX3 were also expressed in the male reproductive tissues and secretions, and have been implicated in male fertility. GPX1 and GPX3 are well represented and characterized and are located in the epididymal epithelia and sperm. These selenoproteins are reported to protect the epididymal parenchyma and maturing sperm from oxidative stress (reviewed in ref. [74]).

The foregoing evidences highlight that Se performs significant functions in the male reproductive system which are regulated by selenoproteins, especially GPX4 and SELENOP. Therefore, it is advisable to perform more studies focusing on the elucidation of additional roles played by these (GPX4 and SELENOP) selenoproteins in male reproductive functions. Nevertheless, search for the elucidation of additional putative mechanisms potentially modulated by other biologically relevant selenoproteins should also be included in the scope of future studies. In any case, selected mammalian selenoproteins (expressed in mouse model) having potential implications in male reproductive function are summarized in Table 3.

## 3. Role of Selenium in Male Reproduction

In past, Behne and colleagues conducted a set of studies to observe Se regulation and its importance in male rats; these authors reported that, in conditions of deficient Se intake its supply to testes was prioritized over other tissues and organs. Subsequently, results of such studies allowed to assume that Se is potentially involved in testosterone biosynthesis [11,81].

### 3.1. Role of Selenium in Steroidogenesis and Spermatogenesis

The optimal concentrations of primary sex hormone (testosterone) are essential for normal development of sperm cells. During the process of testosterone biosynthesis, ROS are generated and their excessive production contributes to male infertility [82]. Interestingly, almost two decades ago, *Selenop* mRNA was selectively identified in Leydig cells of rats [82,83]. It was suggested that, in addition to its role as a plasma selenoprotein transporting Se, Selenop may also play a role as an intracellular antioxidant in Leydig cells [83]. Besides, cytosolic Gpx was also implicated in counteracting the H_2_O_2_ generated as a result of testosterone biosynthesis, but its expression in testis was relatively lower [82]. However, as for the effects of Selenop on steroid biosynthesis in Leydig cells, it was proposed that, in addition to its intracellular antioxidant role, Selenop might also act as an extracellular antioxidant protecting Leydig cells from oxidative damage. Subsequently, in 2001, using the mouse and rat models, a physiological function of Selenop in testosterone production was reported in Leydig cells [82]. It was suggested that Selenop plays an important antioxidant role in protecting Leydig cells from the oxidative damage resulting from testosterone biosynthesis pathway [82,83].

In any case, the growing body of literature also suggests that Se is also important for the biosynthesis of testosterone. It has been suggested that blood testosterone concentrations have positive correlation with concentrations of Se [84,85]. Similarly, dietary Se supplementation has been demonstrated to ameliorate the testosterone level and quality of semen in different animal species [12,86,87,88]. However, the exact underlying mechanisms regulating the testosterone production are yet unclear and require further understanding. Since testosterone is produced by Leydig cells, they could serve as a potential target model to further investigate such mechanisms of Se-related modulation of testosterone synthesis [89]. It has been demonstrated that Se has a potential regulatory role in some essential cellular functions. This role is reportedly modulated by activating the extracellular-signal-regulated kinase (ERK) signaling pathways [90]. However, the exact role of Se in biosynthesis of testosterone via modulation of ERK signaling pathway and the fate of Leydig cells throughout the process of sperm development is incompletely understood [89]. In this connection, in an in vitro study, Shi et al. [89] revealed that Se could modulate the proliferative and apoptosis-related cellular events in ovine Leydig cells. These effects were principally achieved via regulation of oxidative stress, cell cycle, and apoptosis-associated biomarkers. The lowest ROS content and the highest GPX activity were found in the 2.0 μmol/L group compared to the control (without Se) and high Se-treated (4.0 and 8.0 μmol/L) groups. They further reported that Se could also increase testosterone biosynthesis in Leydig cells via activation of ERK signaling pathway and expression of its downstream genes i.e., steroidogenic acute regulatory (*StAR*) and 3 beta-hydroxysteroid dehydrogenase (*3β-HSD*). These biomarkers are thought to have a close relation with the regulatory implications of Se in fertility and gametogenesis in males. Optimal levels of Se (2.0 μmol/L) triggered a significant up-regulation in the expression of these genes and improved testosterone biosynthesis. However, when the concentration of Se was increased (8.0 μmol/L) in culture medium, the proliferative capacity of Leydig cells and the expression of cell cycle-related biomarkers were reduced, and the ratio of cells undergoing apoptosis was also significantly raised. Intriguingly, latter findings were coherent with the relative expression of pro-apoptosis-related markers [89]. Interestingly, maternal dietary Se supplementation (0.5, 2.0 mg Se/kg dry matter (DM)) led to the statistically higher indices related to testes histomorphology, and significantly higher (compared to the controls) density of spermatogenic cell lines and Leydig cells was recorded in testicular parenchyma of young “Taihang Black” male goats [91]. In contrast, these indices (testicular weight and volume) were significantly decreased in Se-excess (4.0 mg/kg) group. Improved levels of testosterone in testicular tissue and serum, and increased expression of testosterone biosynthesis-related biomarkers were observed in Se-treated groups. The relative mRNA expression of *StAR, 3β-HSD* and cytochrome P450 family 11 subfamily A member 1 (*CYP11A1*) was reduced when the level of Se was increased in the maternal diet. Supplementation of Se in maternal diet could also influence the expression of androgenic receptor protein in testes of resulting progeny [91]. Furthermore, in an interesting comparative transcriptomic study on testes of new born bovine calves, Cerny and colleagues [92] reported that the form of Se (35 ppm organic Se, 35 ppm inorganic Se, and 50:50 mix of both (MIX)) fed to cows during gestation differentially impacted the expression of several mRNAs putatively regulating the encoding proteins implicated in steroidogenesis and/or spermatogenesis. Relatively pronounced and desirable transcriptional expression of mRNAs implicated in steroidogenesis and spermatogenesis were observed compared to the calves born to cows fed inorganic Se during gestation [92]. Of note, testes of neonatal calves born to cows fed MIX (50:50 organic and inorganic Se) showed an independent transcriptomic phenotype that was not intermediate between the organic and inorganic groups [92]. It is worthwhile to mention that all claves used in this analysis were of high/adequate Se status and were born to the dams of high/adequate Se status. However, at present, the mechanistic and physiological bases of these differential transcriptomic events are incompletely understood and future well- powered studies with adequate number of animals are awaited. Nevertheless, previously it has been demonstrated that organic Se resulted in the higher blood and liver tissue levels of Se compared to the inorganic supplemented cows [92,93,94].

Similar to its role in steroidogenesis, Se has also been implicated to play an important role in spermatogenesis. In one of the classical studies on the role Se in male fertility and reproduction, Watanabe and Endo [95] examined the morphology of sperm and spermatocyte chromosomes in mice fed Se-deficient diet. They reported that the ratio of abnormal sperm was high (6.8% to 49.6%) in Se-deficient group compared to the control group (4.0% to 15.0%). The morphological defects were more pronounced in sperm head compared to other regions i.e., midpiece and tail. However, the frequency of chromosomal abnormalities in spermatocytes (MI stage) was comparable between Se-insufficient and the control groups [95]. Besides, in another study, Se-deficient (yeast-based Se 0.02 ppm) feed significantly reduced the number of spermatogenic cell line i.e., pachytene spermatocytes, spermatids and maturing sperm in mice [96]. Taken together, these findings corroborate that Se might have strong implication in the process of spermatogenesis in males.

Recently, in an in vitro cell culture study on Sertoli cells of newborn bovine calves, it has been shown that Se supplementation (inorganic Se; 0.25, 0.50, 0.75, and 1.00 mg/L) in culture media could influence the cell viability and expression of essential protein components (occludin, connexin-43, zonula occluden, E-cadherin) of blood–testis-barrier. In fact, this effect of Se was dose-dependent and exhibited the U-shaped response i.e., optimal ameliorative effects were observed at moderate doses (0.25, 0.50), and temporary cytotoxic effects were evident when the dose was increased to 0.75, and 1.00 mg/L [97]. Interestingly, the mechanistic basis for these effects of Se was later elucidated by the same group of researchers [98], where they demonstrated that Se-pretreatment could ameliorate microcystin-LR (MC-LR)-triggered cytotoxicity in bovine Sertoli cells. Pre-treatment with inorganic Se (0.50 mg/L) inhibited the nuclear factor kappa B (NFκB) activation in cultured Sertoli cells. Selenium also exhibited some immunomodulatory roles, which were evident by the inhibition of inflammatory cytokine activation in MC-LR-exposed Sertoli cells [98]. Besides, Se-pretreatment also modulated the expression of mitochondria-related genes such as Cytochrome c oxidase subunit I (*COX-1*), Cytochrome c oxidase subunit 2 *(COX-2),* Acetyl-CoA acetyltransferase 1 *(ACAT1),* Mitochondrial transcription factor A (*mtTFA*) and Subunit 2 of NADH dehydrogenase (*MT-ND2*). Se-treatment also modulated the apoptotic events in Sertoli cells, which were highlighted by the inhibitory effects on apoptosis induction via cytochrome-c release and expression of caspase-3. Mitophagy and mislocalization of components of blood–testis-barrier were also inhibited in Se-treated Sertoli cells compared to the MC-LR-exposed group. Intriguingly, the peculiar antioxidant effects of Se were also observed, which were evident by an increased GPX4 activity in cultured Sertoli cells [98]. Since, NFκB and mitochondrial signaling pathways, and blood–testis barrier (an essential measure for protecting the testicular parenchyma) are implicated in delicate cellular functions in testicular parenchyma, results of these studies reasonably provide the novel and enticing evidence that Se, at least in part, could help ameliorate the Sertoli cells-related perturbations is spermatogenesis and male fertility. Nevertheless, both in vitro and in vivo studies involving other animal models will be interesting, and have the potential to provide concreate evidence with regards to ameliorative roles of Se in Sertoli cell function and spermatogenesis.

There is also an evidence (in vivo) that Se might have an implication in modulating the process of spermatogenesis via regulation of expression of genes related to cell cycle and apoptosis [99,100,101]; however, the precise mechanistic basis for this Se-mediated modulation is still unclear [89]. It has been demonstrated that components (*cjun* and *cfos*) of redox active transcription factor, Activator protein 1 (AP-1), modulate the proliferation and differentiation of cells, and they are also implicated in modulating the signal transduction pathway, and also play a regulatory role in steroidogenesis and spermatogenesis. It has also been shown that the expression of transcription factor (AP-1), *cfos* and *cjun*, is stage specific and increased during the phase of active spermatogenesis [102]. It was reported that when mice were fed a Se-deficient diet (yeast-based diet with 0.02 ppm Se), a significant decline in testis Se-levels was observed compared to those who received a Se-adequate (0.2 ppm inorganic Se) diet, and a significant increase was observed in Se-excess group (1 ppm in organic Se) compared to the Se-adequate and Se-deficient groups. GPX activity and lipid peroxidation (LPO) levels were also decreased and increased, respectively in Se-deficient group. Interestingly, a significant increase was also observed in the mRNA and protein expression of cfos and cjun in Se-deficient mice [102]. Of note, 8-weeks-long Se-deficiency lead to a significant reduction in the number of spermatogenic cells i.e., pachytene spermatocytes, late and mature spermatids. Intriguingly, the effect of Se-deficiency was less intense on germ cell kinetics at the early stages, suggesting a potential implication of Se-deficiency in affecting the overall sperm quality parameters observed in other similar studies [102]. It has been suggested that loss of AP-1 (cJun/cFos) expression during the meiotic stages might have an involvement in preventing the progression of these gem cells to differentiate into spermatids [103]. It has also been demonstrated that Se-deficiency, and to a lesser degree, Se-excess perturb these regulatory processes. As for the impact of high Se levels on modulation of AP-1, it has been suggested that Se at higher levels react with GSH and thiols present in proteins, contributing to the constitution of -S-S- and GS-Se-SG. Since the regulation of Fos and Jun is mediated by the redox status of a critical cysteine residue, thus the oxidation by excessive levels of Se may preclude the binding of cJun homodimers at the promoter sites and may lead towards the decreased *cjun* mRNA [103]. In addition, mitogen-activated protein kinase (MAPK) has been implicated in modulating *cfos* gene expression, and high Se levels have been linked with inhibition of MAPK/c-Jun amino-terminal kinase (JNK) pathways. Therefore, these two mechanisms are suggested to have an involvement in decreasing the expression of *cjun* and *cfos* in condition of excess Se [96]. Conversely, the reduced expression of these transcription factors in limiting Se conditions may be attributed to the increased ROS levels and subsequent oxidative damage at the promoter sites in these transcription factors, since the binding to AP-1 control sequence is reduced in Se-deficient conditions [103].

Similarly, NFκB, a widely known redox-regulated transcription factor reportedly performs an important function in spermatogenesis. It has been shown that an increased relative expression of both *p65* and *p50* genes (components of NFκB) was observed in male mice consuming Se-deficient diet (0.02 ppm inorganic Se) [101]. Besides, the levels of inducible nitric oxide and expression of inhibitory protein IκB were increased and decreased, respectively in Se-deficient mice [101]. Previously, it has also been suggested that during conditions of oxidative stress in testis, NFκB proteins paly a pro-apoptotic role in spermatogenic cells, which elicits the hypothesis that an increased expression of NFκB might lead to an exuberant germ cell death and reduced fertility. Therefore; these findings clearly demonstrate that the decreased Se supply has potential negative implication on reproductive efficiency and spermatogenesis in mice via modulating the expression and activation of NFκB in testes [101]. Intriguingly, it has been demonstrated that Se-mediated oxidative stress also has a potential implication in the modulation of expression of HSP70, HSP70-2, and MSJ-1 (a chaperone partner of spermatogenic cell-specific HSP70-2), contributing to an impaired spermatogenesis and reduced fertility in mice [104]. Previously it has also been demonstrated that the alterations in Se-levels (deficiency or excess) could lead to significantly increased apoptosis (p53-meidated) in spermatogenic cells in mice [100]. Therefore, these findings and others described before lend reasonable insights and add new dimensions to the understanding of underlying Se-mediated molecular mechanisms implicated in impairing the process of spermatogenesis and male fertility. However, the exact mechanisms behind such detrimental effects of alterations in Se-levels are still incompletely understood and require further elucidation.

**Figure 3:**
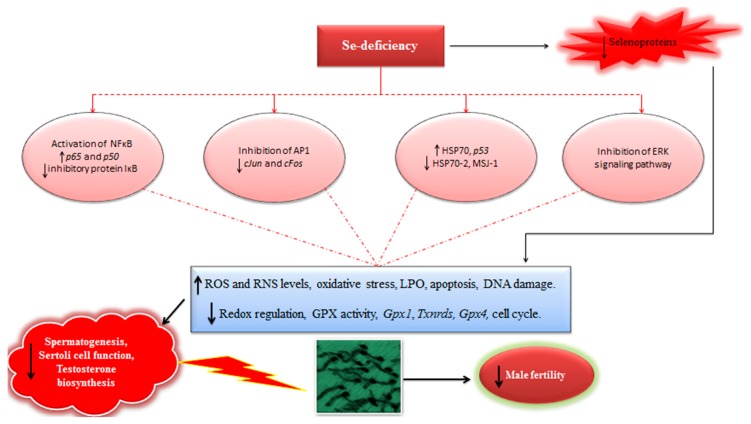
Schematic illustrating the implication of Se-deficiency in steroidogenesis, spermatogenesis, and male fertility (For details see the text in Section 3.1).

In light of available evidences, it could be inferred that Se-deficiency has a strong implication in modulation of redox signaling and intracellular oxidative stress, triggering the expression of several redox-sensitive transcription and proliferation factors, and ultimately affecting the process of spermatogenesis and male fertility (Figure 3). Therefore, well-powered studies focusing on the elucidation of Se-mediated modulation of transcription and proliferation factors should be continued in future. Besides, it is also envisaged that like GPX4 and SELENOP, other selenoproteins may also have certain complementary roles in steroidogenesis and spermatogenesis in males. For instance, in situ hybridization experiments have demonstrated high levels of *Selenov* mRNA in seminiferous tubules in mice, but its exact function in spermatogenesis still remains largely unexplored. Recently it has been shown that selenoprotein U (absent in mouse and human) has a potential implication in modulation of phosphatidylinositide 3-kinases–AKT–mechanistic (or mammalian) target of rapamycin (PI3K–AKT–mTOR) signaling pathway in chicken Sertoli cells (SC) and could perform a novel regulatory role in the SC-mediated spermatogenesis [105]. Therefore, similar studies focusing on the possible physiological and regulatory functions of other selenoproteins such as, selenoprotein K (SELNOK), selenoprotein F (SELENOF), selenoprotein S (SELENOS), SELENOW, and others in mammalian counterparts may also provide enticing results and improve our understanding regarding Se and selenoprotein-mediated modulation of reproductive function in mammalian males. Moreover, it has been reported that seasonal variations could also influence the production of sperm. Therefore, it is highly recommended that replication of these findings in adequately powered studies (involving higher domestic animals) are needed to gain confidence with regards to the interpretation and comparison of evidences currently at hand. Meanwhile, such studies should also focus on an adequate (multiple) number of spermatogenesis cycles for ensuring the optimal effects of Se supplementation on steroidogenesis and spermatogenesis.

### 3.2. Implication of Se on Male Fertility-Related Parameters

It has been reported that sperm maturation process has a strong association with the sperm and ejaculate quality, and overall reproductive efficiency in males. Therefore, any abnormality in such processes might result in ejaculates of inadequate and poorer quality and declined fertility in males [14]. Similarly, increased Se dietary intake has been implicated in enhancing the antioxidant GPX activity, thereby improving the fertility in males [15]. Meanwhile, as discussed before, an excessive supplementation could also result in no therapeutic advantage or even deleterious effects on overall reproductive outcome in males.

It is also worthwhile to mention that, even though the overproduction of ROS results in oxidative stress-induced DNA damage and/or apoptosis, membrane peroxidation and decreased sperm motility, an optimal level is necessary to carry some vital sperm functions viz. capacitation and acrosome reactions [7]. Therefore, a delicate balance in the redox regulation is necessary for the optimal functioning of cells. It has been shown that the alterations in Se-levels may perturb the redox status and could lead to oxidative stress, adversely affecting male fertility by altering the expression of biologically important markers and activity of antioxidant enzymes. To this effect, Kaushal and Bansal [104], reported that the alterations in Se-levels perturb the expression profiles of HSP70 proteins (heat shock proteins) and induce the oxidative stress. When mice were fed Se-deficient (0.02 ppm inorganic Se) and Se-excess (1.0 ppm inorganic Se) diets, a significant increase was observed in the levels of oxidative stress-related markers such as LPO, malondialdehyde (MDA) and free radicals (reactive oxygen species). Besides, a decreased level of GPX was noticed in Se-deficient mice. Conversely, Se-excess group showed relatively higher levels of GPX. Interestingly, the overall fertility-related markers were significantly diminished in both groups [104]. In another similar study, increased LPO, elevated oxidative stress, and reduced levels of GPX were observed in male mice consuming Se-deficient diet [101]. Recently, it has been demonstrated that two months long dietary supplementation of organic Se (0.3 mg per kg body weight) significantly improved the gross morphological and histomorphologcal indices in testes of young male goats. The enzymatic activity of GPX and superoxide dismutase (SOD) in serum and testicular tissue were also significantly ameliorated in young male goats treated with Se compared to the control group [106].

In addition, Stefanov and colleagues [107] studied the effects of organic (1.83g L-SeMet [Sel-Plex] per animal per day) and inorganic (4.0 mg sodium selenite per animal per day) Se supplementation on semen parameters in Bulgarian Merino rams. After 45 days of Se dietary supplementation, improvements were observed in semen volume per ejaculate, sperm motility and overall sperm survival rate in treated rams [107]. Besides, in a very recent study on a mouse model, Asri-Rezaei and colleagues [108] observed that an intraperitoneal injection of sodium selenite (0.50 mg per kg body weight) and Se nanoparticles (0.50 mg per kg body weight) for seven consecutive days adequately improved the tissue Se concentration in testis (as observed on day 28 post-injection). Similarly, the enzymic activities of antioxidant biomarkers were also significantly improved following Se treatment. In addition, the sperm quality parameters such as total count and motility were also improved compared to the control group [108]. In general, these results corroborate that Se supplementation can produce beneficial effects and counter the oxidative stress.

**Figure 4:**
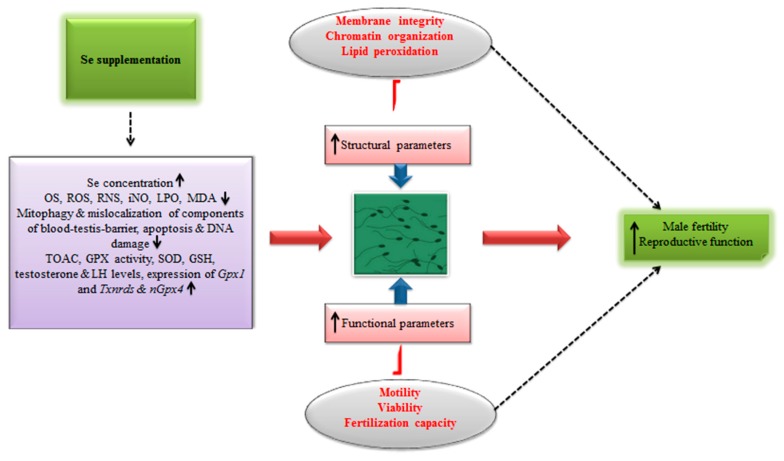
Schematic illustrating the implication of Se supplementation in ameliorating fertility and reproductive efficiency in males.

Besides, recently in an interesting cost–benefit analysis [109], it has been shown that boars fed organic Se-based (0.5 mg per kg) diet produced around 23% higher doses of semen per week compared to those maintained on inorganic Se diets (0.5 mg per kg). A significant increase in revenue (26%) was also observed in this study [109]. In any case, additional animal studies reporting the effects of Se supplementation on the male reproductive efficiency in different animal models are presented in Table 4. Meanwhile, Figure 4 illustrates the implication of Se supplementation in ameliorating the male fertility and reproduction.

It is reiterated that sperm quality parameters such as, sperm concentration, vitality, progressive movement, and overall sperm morphology are regarded as the essential clinical markers for the assessment of reproduction efficiency. Therefore, perturbations in these markers may lead towards sub- and infertility in males. Of note, oxidative stress has been implicated as an important factor contributing towards male infertility [125]. Intriguingly, it is believed that sperm are more sensitive to oxidative damages; this is largely because of the biochemical composition of sperm i.e., it contains higher ratio of polyunsaturated fatty acids and low concentrations of cytoplasmic antioxidant enzymes compared to the somatic cells [126]. Therefore, in conditions of increased ROS-induced oxidative stress, the plasma membrane integrity is affected due to the triggering of LPO cascades. Similarly increased oxidative stress is also implicated in inducing DNA damage in sperm by causing perturbations in chromatin condensation, and the process of chromatin condensation is an essential step for both sperm maturation and fertilization capacity [114]. In this connection, it has been demonstrated that Se deficiency could lead to the impaired chromatin condensation and reorganization processes via elicitation of oxidative stress, and lead to the impaired sperm quality and reduced fertilization capacity in males [114]. Nevertheless, in addition to aforementioned observations of previous studies, further studies focusing on the elucidation of potential markers of DNA integrity will be of high value and should lend encouraging insights with regards to the role of Se in process of spermatogenesis and overall male fertility. Besides, in-depth evaluation of sperm chromatin condensation and reorganization is also biologically more relevant because it is completely reorganized during the later phases of spermatogenesis when histones are substituted by protamines [114]. Further adequately powered studies on Se-deficient models would also improve our understanding in this domain. As demonstrated in previous studies, Se deficiency could impair the sperm quality parameters and antioxidant status in the male reproductive organs, therefore it is reasonable to infer that Se-deficiency potentially perturbs the biosynthesis of selenoproteins having essential roles in redox regulation, leading towards oxidative insult and resultant reduced male fertility parameters. Therefore, in addition to already known mechanisms, more focus should be centered on elucidation of other putative mechanisms implicated in impairing the selenoprotein-mediated redox homeostasis in sperm/ejaculates.

The results of relevant studies with regards to the form of Se used are comparable and encouraging i.e., it is fairly evident that both organic and inorganic forms have the potential to ameliorate male fertility parameters in different animal models (also see Table 4). In order to get a more precise picture, it is also reasonable to conduct more adequately powered studies focusing on the comparative ameliorative effects of different forms of Se. It should also be considered that the effects of Se (especially the organic form) might be impacted by the duration of supplementation; therefore, for obtaining the desirable outcomes with regards to the improved fertility in different animal species, future studies should also include this aspect within their scope. Se-nanoparticles are also reported to possess more bioavailability and less toxicity potential compared to other conventional forms of Se. However, the level of evidence regarding the effects Se-nanoparticles in ameliorating male fertility is still insufficient, and more multifaceted studies are awaited to lend the concrete evidence.

#### Selenium in Seminal Plasma and its Implication in Male Fertility

Seminal plasma is considered to play many essential roles in motility, viability and sustentation of fertilizing ability of mammalian sperm [127]. Villaverde et al. [85] studied the correlation between the serum concentrations of Se and sperm quality traits, testosterone levels, and testes morphology in domestic cats. Se concentrations showed no influence on testosterone levels and sperm production. However, Se concentrations in blood and seminal plasma showed a correlation with sperm quality parameters. It was demonstrated that Se concentrations in seminal plasma were negatively correlated with total testicular weight and sperm morphological traits such as total head defects. Besides, Se concentrations in serum were also negatively correlated with some sperm motility-related parameters including average path velocity, straight line velocity, and curvilinear velocity [85]. These observations, to some extent, highlight that Se (in seminal plasma) may have a potential implication in male fertility and could be used as an important marker in ameliorating the overall male reproductive biology of domestic cats [85]. However, it should be noted that a small number of cats (*n* = 6) was used in this investigation; therefore, inclusion of Se concentration (as a biomarker) in studies focusing on evaluation of potential association between this trace element and sperm quality and fertility in cats should be very cautiously assessed. In addition, for getting reasonable evidences, more data should be gathered from well-powered functional/mechanistic studies using the cat models. Similarly, Bertelsmann and colleagues [128] determined the levels of Se in semen, seminal plasma and sperm, and their association with overall sperm quality and fertility in male horse. It was reported that Se level/concentration in sperm was correlated with sperm quality parameters such as, membrane integrity, progressive motility of sperm, positive acrosomal status and pregnancy rate per estrus cycle. Moreover, Se was also linked with ameliorated sperm quality and fertility in horse. These findings therefore suggest that evaluation of an optimal Se status for equine male reproduction necessitates the analysis of Se in sperm [128].

It is worthwhile to mention that Se in seminal plasma originates from secretion of glandular epithelium of accessory sex glands i.e., prostate gland, seminal vesicles, and epididymis, therefore, alterations in seminal plasma Se-levels indicate that supply of Se to these sex glands changes with dietary Se. It was also demonstrated that effects of Se on sperm motility are likely arbitrated by secretions from these sex glands, either during the process of sperm maturation or at the time of ejaculation [129]. Therefore, it is also reasonable to conduct more research on elucidation of potential functions of Se in seminal plasma and accessory sex glands. This will also improve our current understanding regarding underlying mechanisms and physiological basis of relationship between seminal plasma-Se concentrations and male fertility.

### 3.3. Combinatorial Effects of Se (as a Part of Micronutrient Supplement) on Male Fertility Outcomes (Animal Studies)

Recently, very few studies have demonstrated that Se in combination with other trace minerals and micronutrients can ameliorate the fertility-related outcomes in different animal species. To this effect, in one recent clinical study, Domosławska and colleagues [130] demonstrated that Se-yeast (6 μg per kg) combined with vitamin E (5 mg per kg) supplement (treated for 60 days) significantly ameliorated the semen quality parameters and antioxidant status in clinically healthy dogs with reduced fertility. In Se-supplemented group, significant improvements were observed in parameters such as, blood Se concentration, mean sperm concentration, mean total sperm count, sperm motility, and percentage of live and normal sperm. Furthermore, sperm GPX activity and total antioxidant capacity (TAOC) were also increased significantly [130]. Similar findings were also observed by the same group of researchers in 2015 [131], where the dietary supplementation of Se-yeast (6 μg per kg) in combination with vitamin E (5 mg per kg) for 60 days resulted in the improved sperm quality parameters such as total sperm count, concentration, morphology, and motility scores in clinically healthy dogs with reduced fertility [131]. Similarly, Butt et al. [132] demonstrated that Se (supplemented as 3g Selemax^®^) in combination with vitamin E significantly improved the ejaculate and sperm quality parameters, and increased the concentration of testosterone in Holstein Friesian bulls maintained in a hot and humid environment [132]. Additionally, Ghorbani et al. [133] evaluated the effects of dietary Se (0.3 mg/kg), Zn (40 mg/kg) and their combination on reproductive performance in Sanjabi rams during the breeding season. They reported a significant improvement in sperm concentration, total sperm count, sperm motility following a 120 days long dietary supplementation. However, the concentration of testosterone remained unaltered [133]. In another randomized, double-blinded study, Kirchhoff and colleagues [134] failed to elucidate a clear trend in the extent to which a three months long supplementation of Se/vitamin E (tablets containing 0.1 mg organic Se; capsules containing 100 mg vitamin E) influences the semen qualitative traits in normospermic Cairn Terriers. Nevertheless, an effect of supplementation (head sperm defects) and a substantial interaction between time and treatment was observed for some semen parameters such as, percentage of viable sperm, sperm head irregularities, defective acrosome, and proximal cytoplasmic droplets. Nevertheless, it is worthwhile to mention that the number of dogs (*n* = 9; three dogs per treatment group) used in this randomized study was too low and the subjects included were normospermic i.e., without any apparent semen quality defects. In any case, results of three recent studies (2016–2019) reporting the effects of combination treatment of Se and other micronutrients are summarized in Table 5.

In general, the results of these studies are encouraging and suggest that Se in combination with other essential micronutrients could improve the reproductive efficiency in males. However, the level of concrete evidence is still insufficient and somehow inconsistent. Therefore, well-powered randomized studies will be of high value for building the solid scientific evidence in this regard. It should also be noted that these ameliorative effects of integrated mixture of essential micronutrients might be a result of augmentative and camouflaged effects of each essential micronutrient [135]. Currently, the underlying mechanisms of such combinatorial effects are largely unclear and should be considered in future investigations [43]. Nevertheless, such studies will also lend reasonable basis for determining the proper and adequate remedy protocols aimed at ameliorating male factor sub- and infertility in mammalian species, particularly in domestic and companion animals.

### 3.4. Selenium and Sperm Cryopreservation

Despite significant progress made towards optimizing the cryopreservation technology, confounding factors, and underlying mechanisms related to cryoinjury and freeze tolerance of mammalian sperm remain incompletely understood. Cryopreservation is believed to result in structural and functional losses, and could also lead to apoptosis and reduced fertilization rates. Recently, it has been demonstrated that cryopreservation leads to the differential expression of several mRNAs, long non-coding RNAs, and microRNAs implicated in various essential signaling pathways such as, PI3K-AKT, p53, cAMP, cell adhesion, MAPK, calcium signaling pathways, environment stimuli, apoptosis, and metabolic activities [137,138]. These detrimental effects are linked to the excessive production of ROS and subsequent oxidative insult leading to the overall reduced sperm quality and fertilization rates. Use of antioxidants in semen extenders has been reported to ameliorate these damages and improve IVF outcomes to some extent; however, much remains to be studied in this regard.

One recent study [139] reported that when Se (2 mM) was added in semen extender, sperm quality parameters such as, motility, concentration, and membrane integrity were significantly improved following freezing-thawing of buffalo bull semen. Surprisingly, authors have reported that conception rate was significantly higher (60% vs. 30%) in buffaloes which were subsequently inseminated with Se-treated semen [139]. However, it should be noted that this study seemed to be relatively weakly designed and supporting data do not lend reasonable evidence with respect to conception rate following insemination. Therefore, there is a definitive need to validate these findings in well controlled and properly powered studies in future. In comparison, the study of Khalil et al. [140] seems to be more powered and results are more reasonable. These authors supplemented the semen extender (Tris-yolk fructose) with Se nanoparticles at different doses, and report that 1.0 mg/mL Se supplementation adequately improved the semen and sperm quality parameters including membrane integrity and sperm viability post-thawing in Holstein bulls. Besides, antioxidant biomarkers (in seminal plasma) such as TAOC and MDA were also significantly ameliorated in Se-treated group compared to the control. The rate of apoptosis and necrosis in sperm was also significantly reduced in two Se-treated groups (0.5 and 1.0 mg/mL), however, the lowest rates were observed in the 1.0 mg/mL Se group. Conversely, a higher dose of Se (1.5 mg/mL) resulted in some deleterious effects on sperm quality parameters and increased the ratio of apoptosis in sperm. Interestingly, the conception rate was significantly improved (90% vs. 59%) when cows were inseminated with cryopreserved semen from Se-treated (1.0 mg/mL) group [140]. Albeit, the quality and level of such evidence is still low, these findings, to some degree, support the notion that Se supplementation, particularly in nano-form, could be integrated in sperm cryopreservation protocols, and subsequently, improved fertility rates could be achieved in farm animals following insemination with cryopreserved semen.

## 4. Effects of Se Supplementation on Different Experimentally-Induced Challenges in Male Reproductive System (Animal Model Studies)

There is scarcity of scientific knowledge related to the possible genotoxic effects of inadequate Se concentrations in diet. Recently, Graupner and colleagues [141] studied the genotoxic effects of two-generational Se-deficiency in mice. They investigated the aftermaths of a two-generational Se-deficient diet (0.01 mg/kg) with respect to induced pre-mutagenic DNA damage and mutations in testicular tissue in mice. The life-long Se deficiency was stimulated by providing a Se-deficient diet to mice during two generations viz. starting in the parental generation and continued during fertilization, gestation, postnatally and till the offspring achieved the early adulthood. Authors reported that higher endogenous levels of DNA damage were recorded in testicular parenchyma in mice fed Se-deficient diet compared to those receiving Se-adequate (0.23 mg/kg) diet [141]. These findings, therefore, to some degree, suggest that generational Se deprivation is genotoxic and may have possible entailments for male fertility and de novo mutations presumably carried to offspring, and justify further detailed elucidation. Besides, various reports involving human and animal models have demonstrated that increased levels of varicocele-induced oxidative stress might have potential detrimental effects on testicular parenchyma and semen in males. Recently, Taghizadeh and colleagues [142] demonstrated that dietary supplementation of inorganic Se could ameliorate the experimentally-induced testicular damage in a varicocelized male Wistar rat model. Although, the pathophysiologic bases of varicocele are not clearly understood, the findings of this study provide reasonable evidence that varicocele-induced testicular damage in rats could be ameliorated by supplementation of Se, and these ameliorative effects of Se might be linked to its strong antioxidative and catalytic properties [142].

In recent past, a number of studies evaluating the potential implication of Se in ameliorating the reproductive efficiency in different experimentally-induced models have been reported. Of note, in one very recent study, Gan and colleagues [143] demonstrated that Se-nanoparticles could ameliorate the reproductive efficiency in rats subjected to nickel (Ni) exposure. Se-nanoparticles (0.5, 1.0, and 2.0 mg/kg), when supplemented (oral gavage) for 14 days, adequately ameliorated the Ni-induced testicular damage, DNA perturbations, and testosterone biosynthesis via modulation of phosphorylation of ERK1/2, p38, and JNK-MAPK pathways. The mRNA and protein expression levels of StAR, CYP11A1, and 17β-HSD were also significantly increased in Se-treated rats [143]. Similarly*,* recently Kaur and colleagues [144] reported that Se-treatment (0.5 ppm sodium selenite/kg diet) could improve the Bisphenol A-induced oxidative damage and apoptosis in mouse testes. Compared to Bisphenol A-exposed mice, Se-treatment significantly improved the sperm quality parameters such as, motility and concentration, and reduced the ROS and LPO levels and the rate of apoptosis in testes [144]. An increase in number of spermatogenic cells (number of spermatogonia, spermatocytes, round and elongated spermatids) was also observed in Se-treated group compared to the Bisphenol A-exposed group [144]. In another study on a mouse model [145], zearalenone-induced testicular tissue and sperm damage was adequately alleviated following Se-yeast supplementation (in a dose-dependent manner). Sperm quality parameters such as, concentration and motility were significantly ameliorated in Se-treated mice. The MDA levels were significantly reduced, and levels of enzymatic activities of Gpx and Sod were significantly improved in Se-treated groups compared to the zearalenone-affected group. Similarly, the relative mRNA expression of apoptosis-related genes was also adequately modulated (expression of *Bax* and *Casp3* was reduced, and that of *Bcl2* was increased) by Se treatment. Interestingly, the relative mRNA expression of blood testis barrier-related genes i.e., *Cdh2* and *Vim* was also significantly increased following Se supplementation [145].

These findings and several similar studies (see Table 6) provide reasonable evidence that Se supplementation could ameliorate the reproductive efficiency in animals exposed to different experimental/toxicity-induced challenges. From these evidences it could be ascertained that Se exerts its effects, at least partly, through regulation of antioxidant balance and modulation of certain important molecular mechanism implicated in spermatogenesis and steroidogenesis. Although the results of studies at hand are enticing and encouraging, the volume of these studies is still insufficient to make the solid recommendations. Therefore, future studies focusing on the elucidation of additional putative mechanisms and signaling pathways through which Se might ameliorate the toxicity-induced damages and reproductive efficiency in males should be the center of attention. Results of such studies are anticipated to open new dimensions in the subject of male fertility. In any case, additional recent studies focusing on the effects of Se supplementation on different experimentally-induced challenges are summarized in Table 6.

## 5. Human Studies (Clinical Evidences)

In extant literature, few clinical trials elucidating the effects of antioxidant supplementation on male fertility are available. However, due to inconsistencies in enrollment of subjects and heterogeneous designs, the comparison of such studies is complicated [7,155]. In a recent systematic review on the effects of antioxidant therapies on male sub-fertility, Smits and colleagues [156] concluded that subfertile couples should be advised that evidence at hand regarding the use of antioxidants is indeterminate and inconsistent, primarily because of low event rates and small overall sample sizes, poor design and inadequate methods of randomization in those studies. Such studies have also failed to report key clinical outcomes such as live birth rate and clinical pregnancy. Therefore, higher numbers of large properly-designed randomized placebo-controlled trials focusing on true clinical outcomes such as pregnancy and live births are still needed to clearly understand the exact implication of antioxidants in ameliorating male sub-fertility. Intriguingly, in one of the largest adequately powered clinical trial of its kind with a “well characterized” study population conducted in the USA, Steiner and colleagues [157] demonstrated that combinatory antioxidant therapy (containing Se 200 μg, for details see Table 7) for three months does not improve the semen parameters and clinical outcomes in 174 couples suffering from male factor infertility [157]. These authors also demonstrated that several of the past clinical studies/trials in which antioxidant therapy has been reported to improve the sperm parameters have been limited by a small number of participants, heterogeneity in subjects, variety of antioxidants, and non-clinical endpoints. Authors further argue that their study provides a relatively stronger evidence base compared to previous studies. In any case, here we briefly review some of the relevant reports focusing on the implication of Se (supplemented alone or in combination with other micronutrients) in male fertility and sperm parameters.

In 28 Turkish men with idiopathic infertility, sperm were reportedly under excessive oxidative insult, albeit, the semen quality parameters were ascertained to be at par with the World Health Organization criteria. Along with the increased ROS-related DNA damage in sperm, the levels of MDA, protein carbonyl group and nitrotyrosine were increased in the idiopathic infertile males [158]. These elevated oxidative stress parameters observed in seminal plasma of men with idiopathic infertility also indicate that their reproductive organs were unable to counter the oxidative stress. Thus, it may, very likely, add to the reduced reproductive capacity of subjects suffering from idiopathic infertility [158]. Recently, it was reported that Se-treatment (5 μg/mL of sodium selenite) significantly improved the sperm quality and viability in cryopreserved semen obtained from 42 clinically healthy Iranian men [159]. Percentage of sperm with normal morphology and motility was adequately improved in Se-treated group compared to the untreated control group. A reduced frequency of post-thaw DNA damage was also evident in sperm [159]. Similarly, in another recent study involving 50 asthenoteratozoospermic men, Ghafarizadeh et al. [160] reported a significantly higher score for sperm quality parameters such as motility, viability, and mitochondrial membrane potential in the test group (treated with 2 μg/mL Se at 37 °C for two, four and six hours). Significantly lower levels of MDA and DNA fragmentation were observed in Se-supplemented group. These authors concluded that in vitro Se supplementation may shield sperm from harmful effect of ROS during sperm sampling by keeping the enzymic and antioxidative defense mechanisms in optimal conditions [160]. These findings are also supported by previous findings of Scott et al. [161], who reported that sperm motility was significantly enhanced in a group of sub-fertile men following supplementation with 100 µg Se, daily, for three months. Among those who received Se supplementation, 11% of men attained paternity as compared with none in the placebo group [161]. Nevertheless, high Se supplement (around 300 μg per day) has also been reported to reduce sperm motility by 32% in 11 healthy men. Even though this reduction in sperm motility does not inevitably foretell the declined fertility, the raising frequency of Se supplementation in the healthy population evokes the need for larger adequately powered studies to fully elucidate this potential side effect [129]. In addition to aforementioned evidences, we have summarized (Table 7) the findings of major clinical trials (2009–2019) investigating the role of Se in sperm parameters and fertility in men. It is worthwhile to mention that the only study (excluding a recent study on combination effect of Se) without any positive Se supplementation effect (300 μg/d Se-yeast vs. placebo; [162]) was the one conducted in the USA, where there is no widespread Se deficiency. Nevertheless, considering the differences in intake of Se in different demographic locations around the world, and the variations in soil Se levels and differences in Se content in food regime within individuals, and other confounding factors, it would be important to conduct more of well-powered multi-center randomized intervention trials (vs. control) in larger population cohorts of known Se deficiency. However, before conducting such randomized trials, type and duration of Se supplementation (with or without other antioxidant micronutrient) should also be carefully determined and optimized. Such carefully conducted trials have the actual potential to give us the clear picture with regards to the notion that whether Se supplementation at increasing doses should be encouraged for improving fertility outcomes in men. In any case, that U-shaped response of Se should always be carefully considered while designing the dietary Se supplementation trials in men. 

## 6. Discussion and Perspective

This review spotlights the implications and possible roles of Se and selenoproteins in overall male reproductive capability and related insufficiencies in both animal and human models. The sustentation of physiological Se concentration by an optimal diet, or instead through Se supplementation; is an essential requirement not only to ameliorate the reproductive efficiency in both male and female, but also to safeguard the general animal and human health.

It is evident from observations of past studies (both animal and human) that Se is essentially required for spermatogenesis and male fertility, presumably because of its vital role in modulation of antioxidant defense mechanisms and other essential biological pathways and redox sensitive transcription factors. In the realm of males, there is a growing body of literature elucidating the potential molecular mechanisms, and the relevant evidences suggest that Se has a structural role in sperm which is mediated by a peculiar and canonical selenoprotein i.e., GPX4, and has bearings on sperm motility, chromatin integrity, and fertility rate. In addition, adequate transport of Se for synthesis of certain selenoproteins in testes is very important for proper spermatogenesis and steroid biosynthesis; hence, its deficiency or superfluous supplementation may halt the normal process of spermatogenesis, and at large, the overall reproductive efficiency, and possibly might lead towards infertility in males. However, bearing in mind the evidences from mainstream literature, it is also advisable to perform more studies focusing on the elucidation of additional roles played by two peculiar and canonical selenoproteins i.e., GPX4 and SELENOP in male reproductive functions. In addition, search for the elucidation of additional putative mechanisms potentially modulated by other biologically relevant selenoproteins is equally important and should be included in the scope of future studies. For instance, recently it has been demonstrated that selenoprotein U (absent in mouse and human) has an implication in PI3K–AKT–mTOR signaling pathway in chicken SC and could perform a novel modulatory role in spermatogenesis process [105]. Similar studies focusing on the putative regulatory roles of other selenoproteins (i.e., SELNOK, M, S, V, W, and others) in mammalian counterparts should also lend enticing discoveries and findings which will aid more in the repository of biological roles of Se and selenoproteins and their association with male reproductive function in mammalian males.

As for the implication of Se in fertility and reproduction in men, the findings of a handful of clinical trials also support the implication of Se supplementation in ameliorating the reproductive insufficiency in men. Nevertheless, the quality and size of such studies are not sufficient and inconsistent to draw solid conclusions; therefore, more research is awaited for further validating these findings and outlining any therapeutic schemes (Se supplementation) for improving male fertility. Intriguingly, even though Se deficiency is rarely diagnosed in infertile couples, the circumstantial evidence indicates that it would make sense to conduct more diagnosis of Se status. Therefore, search for the reliable and robust diagnostic biomarkers should be extended, which will facilitate the physicians in diagnosis of Se deficiency-related infertility in men. Nevertheless, considering the bell-shaped response curve [168] and potential adverse effects, as observed in “selenium and vitamin E cancer prevention trial (SELECT)”, one should be very careful in determining the optimal dosage of Se; particularly, the subjects with adequate or high Se status must be assessed with the great care.

Besides, the results of studies focusing on evaluation of potential implication of Se in different experimentally-induced/toxicity-related conditions and their detrimental effects on male reproductive efficiency and those elucidating the possible genotoxic effects of low dietary Se on fertility of offspring are also enticing, but volume of such studies is still very low, therefore, thorough consideration is required in future. To sum up, there are several questions related to Se biology in animal and human reproduction which still remain unanswered. A thorough consideration of these scientific questions as well as further understanding of possible functions of Se and selenoproteins should lend further explanations regarding possible implication of Se in male reproduction capability and health.

## Figures and Tables

**Table 1:** Brief description of selenium forms.

Species	General Description [27]	Relevant Literature
Selenomethionine	It is a Se analogue of amino acid, methionine; this form is typically obtained from plant sources (in particular the cereal grains), Se yeast, and other Se supplements. Also reported in animal foodstuffs. It is inserted non-specifically into body proteins in position of methionine. The supplements containing selenomethionine, therefore, are considered to have more bioavailable Se.	[18,26,27,28,29,30,31,32,33,34,35,36]
Selenocysteine	This form is found in animal foods (from their selenoproteins) and is Se analogue of the cysteine (amino acid).	[18,26,27,30,32,33,36]
Selenoneine (2-selenyl-*N*α,*N*α,*N*α-trimethyl-L-histidine)	This is a newly disclosed major Se species in fish i.e., tuna and mackerel; however, at lower levels it is found in squid, tilapia, pig, and chickens. This form possesses strong radical-scavenging activity.	[27,37]
*Se*-methyl-selenocysteine and γ-glutamyl-*Se-*methyl-selenocysteine	These are also obtained from plant sources viz. Se-enriched yeast, garlic, onions, and broccoli. Generally considered as detoxification products, particularly formed in Se-accumulators and plants of the *Brassica* and *Allium* families. It is metabolized to methyl selenol, which is believed to possess anti-tumor effects. No bioavailability data exist for these [18].	[18,26,27,35,38,39]
Sodium selenite and selenate	The components of dietary supplements; selenate at times appears in water supplies. Some selenate is found in plant sources (cabbage) and fish.	[18,26,27,32,33,35,36,40,41,42]

**Note:** A range of advantages of organic Se over inorganic Se are comprehensively catalogued by Surai and Fisinin [21].

**Table 2:** Recommended dietary allowances (RDAs) of selenium for humans and various animal species (values adapted from Qazi et al. 2018 [43]).

Species	RDA
Adult men	M: 55 µg/d *; F: 55 µg/d *
Sheep and Goat	100–200 µg/kg dry matter of feed/d
Pig	150–300 µg/kg dry matter of feed/d
Horse	100 µg/kg dry matter of feed/d
Donkey	150 µg/100 kg BW
Dairy cow	100 µg/kg dry matter of feed/d
Beef cow	300 µg/kg dry matter of feed/d
Bovine calf	100 µg/kg dry matter of feed/d
Camel	400–800 µg/d

* Healthy individuals (age: >18 Y); data for United States and Canada. Further details on RDAs of Se for humans can be obtained from ref. [22]. M: Male; F: Female; BW: Body Weight; d: Day. Y: Years.

**Table 3:** Brief description of known mammalian selenoproteins relevant to male reproduction.

Selenoprotein Gene *	Symbol [12]	General Description/Function [8,27,43,44,47,56]	mRNA *	Protein *	Relevance to Male Reproductive Function
Glutathione peroxidase 4	*Gpx4*	Detoxification of lipid hydroperoxides, Antioxidant in membranes, functions as structural protein in sperm, also implicated in apoptosis	++++	++++	Structural protein of sperm midpiece mitochondrial sheath and involved in sperm chromatin condensation [75]. Implication in male fertility [68].
Thioredoxin-glutathione reductase	*Txnrd3* (TGR)	Part of the thioredoxin system, Antioxidant function, redox regulation, cell signaling	+	+	Implicated in formation of disulfide bond and sperm maturation process [76]. Expressed in post-pubertal testis, particularly abundant in elongated spermatids at the site of mitochondrial sheath formation [75].
Selenoprotein P	*Selenop*	Primarily responsible for Se transport and also performs antioxidative role. Considered as a major contributor to plasma Se and a reliable biomarker of Se status. Its deficiency causes infertility characterized by abnormal sperm in mice	+	+	Implicated in male fertility [77,78]. Implicated in transport of Se to spermatogenic cells [71]. Essential for sperm development in mice [79].
Selenoprotein V	*Selenov*	Largely unidentified, potential role in redox regulation	+	n.d.	Specifically expressed in rodent testes [80]. In situ hybridization trials have demonstrated the expression of *Selenov* mRNA in seminiferous tubules in mouse, however, its precise function in spermatogenesis is largely unexplored [48,80].
Selenoprotein W	*Seleno* *w*	Antioxidant protection	+	+	n.d. *
Selenoprotein K	*Selenok*	Possible antioxidant protection in cardiomyocytes, Endoplasmic reticulum transmembrane protein	++	n.d.	n.d. *
Selenoprotein F	*Selenof*	Role in cell apoptosis and mediation of chemo-preventive effects of Se	+	n.d.	n.d. *
Selenoprotein S	*Selenos*	Cellular redox balance, Possible influence in inflammatory response	+	n.d.	n.d. *
Selenophosphate synthetase 2	*Sephs2*	Required for biosynthesis of selenophosphate, a precursor of selenocysteine, and thus for selenoprotein synthesis	+	n.d.	n.d.

n.d.: not defined. Relative expression levels in mouse testis: ++++, very high; ++, modest; +, low. Note: The contents tagged with “*” are adapted by permission from “Springer Nature Customer Service Centre GmbH: Springer Nature, New York, USA” by [75].

**Table 4:** Animal studies reporting the effects of selenium supplementation on male reproductive efficiency.

Model	Treatment	Key Observations Reported	Ref.
Sprague-Dawley rats	Se nanoparticles at supranutritional levels (0.2, 0.4, or 0.8 mg Se per kg body weight)	Sperm parameters such as, sperm concentration, motility, and morphological features were all improved at supranutritional levels. However, these parameters were significantly affected when rats were supplemented with higher levels (nonlethal level) of Se nanoparticles i.e., at 2.0, 4.0, or 8.0 mg Se per kg body weight.	[110]
Sprague-Dawley rats	Treated with inorganic Se [0.01(deficient); 0.25 (adequate); 3 (excess); or 5 (excess) mg per kg] for four weeks	The U-shaped response of dietary Se was observed on DNA damage and sperm quality. Se deficiency showed a lower expression of sensitive antioxidant selenoproteins (*Gpx1* and *Txnrds*). However, excessive doses of Se impaired sperm quality and this was linked with reduced mRNA expression of *nGpx4*.	[111]
Mouse	Se-supplement (inorganic Se (0.3 μg/g Se) or organic Se-enriched probiotics (containing 0.3 μg/g Se) given for 75 days	Organic Se co-supplemented with probiotics significantly improved male fertility in mice. The ameliorated fertility index included the parameters such as, reduced testicular tissue injury, increased levels of serum testosterone, and improved sperm indices in Se-supplemented group. As such, these improved fertility-related parameters were ascribed to be the result of the antioxidant function of Se.	[112]
Mouse	0.2 ppm sodium selenite; 1.0 ppm sodium selenite	Mice in both groups showed an increased occurrence of mitochondria- and plasma membrane-related defects, and DNA damage in sperm. However, these damages were more pronounced in mice exposed to Se-deficient feed.	[113]
Mouse	Se-deficient diet (0.02 ppm) Se-sufficient (0.2 ppm); organic Se	Sperm from Se-deficient mice demonstrated vitiated chromatin condensation, declined in vitro fertilization ability and increased lipid peroxidation (LPO) in both testes and sperm compared to the Se-sufficient mice.	[114]
Mouse	Se-deficient (0.02 ppm) Se-excess (0.2 ppm); yeast-based Se. Mice were fed for 4 months	Se concentration and GPX activity (in testis) were significantly reduced. The fertility percentage and size of litter were both reduced in Se-deficient group.	[96]
Aged mice	Inorganic Se 0.2 mg/kg body weight	Improved sperm parameters and increased expression of *CatSper* genes were observed in Se-treated group.	[115]
Rabbit	Treated with Se nanoparticles (400 μg/kg) for 60 days	Improved serum testosterone levels were recorded in Se-treated group compared to the control. Besides, improved ejaculate volume and sperm quality parameters such as, sperm morphology, viability were observed.	[116]
Ram	0.5 ppm organic Se; 0.2 ppm organic Se	A significantly higher concentration of Se and improved ejaculate and sperm quality were observed in seminal plasma of rams exposed to a feed containing 0.5 ppm organic Se compared to those who received 0.2 ppm organic Se.	[117]
Boar	Organic Se (0.2 mg per kg); Inorganic Se (0.2 mg per kg)	Ejaculate quality and sperm parameters were significantly improved in boars following dietary supplementation of organic Se (0.2 mg per kg) compared to those treated with sodium selenite at the same dose.	[118]
Aardi buck	Sodium selenite 0.1 mg/kg, Sodium selenite 0.05 mg/kg	Improved sperm count and motility was observed in both Se-treated groups. However, relatively better outcomes were observed in 0.1 mg/kg group.	[119]
Boar	0.5 ppm organic Se	Following 11 weeks of feeding trail, organic Se supplementation increased glutathione peroxidase 4 (GPX4) activity (raw semen) and number of seminal doses in boars.	[120]
Boar	0.3 ppm organic Se; 0.3 ppm inorganic Se	Following 12 weeks of Se supplementation, Se content and GPX activity were increased in semen of boars treated with organic and inorganic Se. Besides, semen quality parameters namely semen concentration and progressive motility of sperm were improved compared to the control group without Se. Improved resistance of liquid stored semen to hypo-osmotic shock and thermal tests, and improved fertility rates were observed in semen of boars treated with Se. All mentioned indices were slightly higher in the organic Se group compared to the inorganic group.	[121]
Buffalo bulls	10 mg organic Se/animal twice a week; and 10 mg inorganic Se/animal twice a week	Three months long Se supplementation significantly improved the sperm quality parameters (ejaculate volume, sperm motility, concentration, and morphology) in buffalo bulls. Besides, testosterone concentrations were also increased in Se-treated groups.	[122]
Saanen bucks	Inorganic Se 0.34 mg/kg body weight supplemented at ten-day intervals for three months	Se supplementation improved the testicular biometry and sperm parameters. GPX activity, plasma testosterone and LH levels significantly increased in Se-treated group from days 40 to 80 compared to the control group. These indices reached peak reached peak at day 80 of the trial.	[123]
Bovine bull	In vitro fertilization (IVF) medium supplemented with Se (100 ng/mL)	A significant increase in sperm mitochondrial activity was observed after 1 h of incubation in Se-supplemented IVF medium. Moreover, Se supplementation after 2 h of incubation showed an increase in HOST-positive (hypo-osmotic swelling test) sperm and sperm acrosome integrity. Increased number of sperm bound to zona pellucida (ZP) was observed in Se-treated group compared to the control.	[124]

**Table 5:** Recent animal studies reporting the implication of selenium supplementation (in combination with other micronutrients) on male fertility outcomes.

Animal Model and Number	Treatment Regime and Duration	Key Findings	Ref.
Male CD-1 mice (*n* = 12 per experimental group)	Fertilix^®^ (CellOxess, Princeton, NJ, USA) was supplemented for two months. (Se 55 μg, zinc 7.5–11 mg, Full spectrum natural vitamin E 104–290 mg, Lycopene 7.5–15 mg Carnitine blend 200–800 mg Folic acid 400–500 mg Vitamin C 30–90 mg).	Eight weeks long pretreatment with the antioxidant formulation completely protected oxidative stress-induced DNA damage in *Gpx5* KO mice sperm. In mouse models of scrotal heat stress, only 35% (19/54) of female mice became pregnant resulting in 169 fetuses with 18% fetal resorption (30/169). Conversely, in antioxidant pretreated group 74% (42/57) of female mice became pregnant, resulting in 427 fetuses with 9% fetal resorption (38/427).	[126]
Four infertile male dogs with low blood Se levels (86.0–165.0 μg/L)	Organic Se 0.6 mg/kg and vitamin E (5 mg/kg) orally supplemented for 60 days.	Treated dogs showed improved sperm parameters. Increase in blood Se concentration (401 μg/L) was observed at the end of trial. When these dogs were used for matting purpose, bitches successfully conceived and gave birth to 4–6 pups.	[136]
Sixteen healthy normospermic dogs (two patients were excluded after adaptation period)	A supplement comprising of Se 0.27 mg/kg vitamin E 250 mg/kg, vitamin B9 1.5 mg/kg, zinc 180 mg/kg, and n-3 PUFA 0.5%, given for 90 days.	In treated group, sperm quality parameters i.e., total sperm count, concentration, sperm vitality and membrane integrity were significantly improved compared to the control group.	[135]

**Table 6:** Studies demonstrating the implication of selenium in ameliorating reproductive efficiency in males exposed to different experimental/toxicity challenges.

Model	Experimental Condition/Treatment Regime	Relevant Results	Ref.
Rats	Se nanoparticles (0.2 and 0.5 mg/kg/d) supplementation ameliorated developmental testicular toxicity induced by maternal exposure of di-n-butyl phthalate (DBP) in Pre-pubertal male rat offspring. Note: Pregnant female rats treated from gestation day 12 to postnatal day 14 day with two doses of Se-nanoparticles (0.2 and 0.5 mg/kg/d) against developmental testicular toxicity induced by DBP (500 mg/kg/d).	Maternal Se treatment significantly increased mRNA expression of *Gpx* and *Sod*, *Insl3,* and *Mr* in pre-pubertal male rat offspring. Malondialdehyde (MDA) and GSH levels were also significantly reduced and increased, respectively in testicular tissue. Besides, histological assessment revealed that damage in testicular parenchyma was also ameliorated.	[146]
Wistar rats	Cadmium-exposed rats treated with Se (0.35 mg per kg body weight) for 28 days.	The activities of testosterone biosynthesis-related and antioxidant enzymes, levels of steroid hormones, and testicular Se levels were adequately ameliorated compared to the Cd-exposed rats. In addition, Se treatment alleviated, at least partly, Cd-induced damage to architecture of testis in rats. Se-treatment also modulated the key testicular injury-related marker enzymes including LDH, SDH, G6PD, G6Pase, ACP, ALP, and AST.	[147]
Kunming mice	Aflatoxin B1-Exposed mice treated with inorganic Se (0.2 and 0.4 mg/kg) for 45 days.	Se-treatment at both doses (0.2 and 0.4 mg/kg) significantly ameliorated the sperm quality parameters such as, morphology, concentration and motility compared to the aflatoxin B1-exposed group. Levels of reactive oxygen species (ROS), MDA were significantly decreased, and activity of Gpx was improved. The level of serum testosterone and protein expression of testosterone synthesis enzymes StAR, P450scc, and 17β-HSD were significantly improved in Se-treated groups.	[148]
Albino rats	Oral deltamethrin-exposed rats treated with combinatory supplementation of Se and vitamin E (1.2 mg/kg body weight Viteselen^®^, containing 1.67 mg sodium selenite + 150 mg vitamin E/mL).	Se treatment significantly ameliorated the sperm quality characteristics, improved the levels of testosterone and testicular GSH, and reduced MDA levels. Similarly, Se-treated group showed markedly improved spermatogenesis and histo-architecture of testis parenchyma compared to the deltamethrin-exposed group.	[149]
Rats	Streptozotocin-exposed diabetic rats treated with Se nanoparticles (0.1 mg per kg body weight).	Se-treated group showed improved antioxidant status and serum testosterone levels. Expression of apoptosis-related genes i.e., *Bax* and *Bcl-2* was also significantly altered. Histological assessment revealed that Se-treatment significantly ameliorated the testicular damage caused by streptozotocin exposure, which was evident by an increased number of spermatogenic cells in the seminiferous parenchyma of rats.	[150]
Wistar rats	Enrofloxacin-exposed rats treated with supranutritional Se (dose not reported by authors) for 21 days.	Se co-administration moderately improved the activity of antioxidant enzymes in testicular tissue and reduced the levels of LPO. Sperm parameters such as, total count, viability were also partly improved.	[151]
SD rats	Nickel sulfate-exposed rats treated with Se-nanoparticles (0.5, 1, 2 mg Se/kg body weight) for 14 days.	Se-treatment adequately alleviated testicular damage in Ni-exposed rats. GPX activity was improved MDA levels were reduced in testes. Besides, the rate of apoptosis was significantly decreased in Se-treated group compared to the Ni-exposed rats. A significant decline was observed in caspase-3 positive cells. Se-treatment significantly decreased mRNA and protein expression of *Bak*, *cytochrome c,* and *caspase-9* in the testis, and increased the expression of *Bcl-2*. These effects were more pronounced in rats treated with higher doses (2 mg) of Se.	[152]
SD rats	Aroclor 1254-exposed rats treated with Se (1 mg Se/kg) Both control and Se-deficient rats were used in this study.	DNA damage was more pronounced in Se-deficient rats exposed to Aroclor 1254. Se supplementation significantly ameliorated DNA damage in sperm in both normal Aroclor 1254-exposed and Se-deficient rats.	[153]
NMRI mice	Dexamethasone-treated mice treated with Se (0.3mg/kg) for 7 days.	Se-treatment increased the mRNA expression of *Catsper1* and *Catsper2* in testes. Improvements were also observed in serum levels of LH. It should be noted that *Catsper1* and *Catsper2* are implicated in important sperm functions.	[154]
Wistar rats	Experimentally varicocelized male rats supplemented with inorganic Se (0.05, 0.1, 0.2, and 0.4 mg per kg body weight).	Sperm quality parameters, antioxidative status were significantly ameliorated, and damage to histo-architecture of testes was significantly lower, and Johnsen’s score was also adequately improved compared to the varicocelized control rats.	[142]

**Abbreviations:** Lactate dehydrogenase (LDH), sorbitol dehydrogenase (SDH), glucose-6-phosphate dehydrogenase (G6PD), glucose-6-phosphatase (G6Pase), acid phosphatase (ACP), alkaline phosphatase (ALP), aspartate aminotransferase (AST), Insulin-like growth factor-3 (*Insl3*), mineralocorticoid receptor (*Mr*), Sprague-Dawley (SD).

**Table 7:** Human studies (2009–2019) involving selenium supplementation to improve male fertility/reproductive status/clinical outcomes.

Condition	Study Type and Location	No. of Subjects and Age	Type and Duration of Treatment	Key Results	Reference
Subjects diagnosed with varicocele and underwent sub-inguinal varicocelectomy	Randomized, single blind clinical trial (intervention vs. control) (Iran)	*n =* 60 infertile men Age: not reported	Oral supplementation of Se (200 ug), Folic acid (5 mg) and vitamin E (400IU) (6 months)	Sperm parameters were improved compared to the control group.	Zadeh et al. [163]
Men with male factor infertility	Multi-center, double blind, randomized, placebo-controlled trial conducted in eight American fertility centers (USA)	*n =* 174 couples Age of males: not reported	500 mg vitamin C, 2000IU vitamin D3, 400IU vitamin E, 1 mg folic acid, 20 mg zinc, 200 μg Se, and 1000 mg L-carnitine (3 months)	No improvements were observed in semen quality parameters or DNA fragmentation. No improvements were observed in conception rate (in vivo).	Steiner et al. (2018) [157]
Infertile men	Longitudinal study (Iraq)	*n =* 12 Age: not reported	50 μg Se (3 months)	Improved sperm count, motility, viability, sperm morphology, and ejaculate volume.	Mossa et al. (2018) [164]
Infertile patients with idiopathic astenoteratozoospermia	Prospective open-label study (Italy)	*n =* 114 (96 completed the study) Age: 21–46 years	Combination treatment including Se 50 mcg + L-carnitine 145 mg + acetyl-L-carnitine 64 mg + fructose 250 mg + citric acid 50 mg + coenzyme Q10 20 mg + zinc 10 mg + ascorbic acid 90 mg + cyanocobalamin 1.5 mcg + folic acid 200 mcg (4 months)	Improvements were observed in sperm parameters such as progressive motility and treatment was well tolerated. Whereas 16 patients achieved pregnancy during the study.	Busetto et al. (2012) [165]
Chronic prostatis	Prospective open-label study (Italy)	*n =* 60 Age: 30–55 years	Se 82.3 μg + lycopene (1.5 mg) + epigallocatechin gallate (250 mg) + ellagic acid (250 mg) + zinc (20 mg) (30 subjects) vs. No treatment (30 subjects) (6 months)	Improved sperm quality parameters (motility and morphology) were observed. Improvements were observed in leucocytospermia and Chronic Prostatitis Symptom Index.	Lombardo et al. (2012) [166]
Idiopatic asthenoteratospermia	Prospective single-arm study (Iran)	*n =* 690 Age: 20–45 years	200 μg/d L-selenomethionine + 400 IU/d Vit E (3 months)	Improved sperm motility, morphology and pregnancy rate were observed.	Moslemi and Tavanbakhsh (2011) [167]
Healthy men	Double blind RCT (USA)	*n =* 42 Age: 18–45 years	300 μg/d Se-yeast or placebo (11 months)	No effects on seminal parameters were observed.	Hawkes et al. (2009) [162]
Idiopatic asthenoteratospermia	Doubleblind RCT (Iran)	*n =* 468 Age: 25–48 years	200 μg Se/d (116 subjects), Or 600 mg NAC/d (118 subjects), Or 200 μg Se+ 600 mg NAC/d (116 subjects) Or Placebo (118 subjects) (6 months)	Improved sperm count, motility and morphology were observed (both in Se + NAC and Se alone groups).	Safarinejad and Safarinejad (2009) [62]
